# Giorgio Agamben (2021) An welchem Punkt stehen wir? Die Epidemie als Politik: Turia+Kant, Wien, 155 Seiten, 16,00 €, ISBN 978-3-85132-996-4

**DOI:** 10.1007/s00481-021-00676-5

**Published:** 2021-11-25

**Authors:** Lukas Johrendt

**Affiliations:** Lehrstuhl für Ethik und Hermeneutik, Humboldt-Universität zu Berlin, Berlin, Deutschland

Das hier zu besprechende Werk ist in einem überaus ambivalenten Sinn ein kritisches: Es ist kritisch in dem Sinne, dass es fundamentale Kritik an den Maßnahmen zur Bekämpfung der COVID-19-Pandemie artikuliert; aber auch in dem Sinne, dass die in ihm vorliegenden zwanzig unterschiedlich langen und in ihrer Entstehung wie in ihrer Gattung divergierenden Texte – neben Zeitungsbeiträgen, Interviews und Aphorismen finden sich auch knappe Essays – des italienischen Philosophen Giorgio Agamben nicht anders als kritisch besprochen werden können. „An welchem Punkt stehen wir?“ ist eine kritisierende wie ebenso streng zu kritisierende Textsammlung.

Giorgio Agamben kann mit guten Gründen als einer der einflussreichsten Protagonisten der gegenwärtigen politischen Philosophie bezeichnet werden. Sein neunbändiges Hauptwerk „Homo Sacer“ (Agamben 2017) liefert eine genealogische Rekonstruktion der Souveränität und muss gleichzeitig als eine radikale Macht‑, Staats- und Gesellschaftskritik gelesen werden. Die in Agambens Werk zentralen Begriffe der „Biopolitik“, des „nackten Lebens“ und des „Ausnahmezustands“ sind auch in „An welchem Punkt stehen wir?“ an entscheidenden Stellen wiederzufinden. Insofern stellt die vorliegende Textsammlung eine Aktualisierung und eine situationsgeleitete Radikalisierung der Theorie Agambens dar.

Michel Foucault fasste seine Antwort auf die Frage „Was ist Kritik?“ in einem Satz zusammen: „Die Kunst nicht regiert zu werden bzw. die Kunst nicht auf diese Weise und um diesen Preis regiert zu werden“ (Foucault 1992, S. 12). Agambens Überlegungen zur COVID-19-Pandemie lassen sich in eben dieser Perspektive kritisch nennen. Hier schreibt ein politischer Philosoph, der nicht auf diese Weise und besonders nicht um diesen Preis regiert werden will. Eben hier liegt die anzuführende Kritik: Ist der Preis wirklich derjenige, den Agamben in seinen Texten behauptet? Wird hier wirklich auf die von Agamben angeführte Weise regiert? Kurz: Existiert der Gegenstand der Kritik?

Besonders fraglich scheint die Existenz des kritisierten Gegenstands in einem der frühesten Texte der Sammlung. In „Die Erfindung einer Epidemie“ beschwört Agamben nichts weniger herauf als „eine vermeintliche Epidemie“ (S. 15). Diese sei von Regierungen weltweit erdacht, da das bisher auf einer mehr oder weniger abstrakten Terrorismusgefahr beruhende Regierungsparadigma des „Ausnahmezustands“ ausgedieht habe. Um den zur Regel gewordenen Ausnahmezustand aufrecht erhalten zu können, müsse nach der terroristischen nun eine virale Gefahr erdacht und so Grundrechte ausgehebelt werden, um „souverän“ regieren zu können (S. 16–18). Auch wenn Agamben selbst im weiteren Verlauf des Bandes nicht mehr von der „Erfindung einer Pandemie“ spricht, so bleibt doch jene grundlegend kritische Haltung bestehen. Werden hier bewusst Freiheitsrechte zur Macht- und Souveränitätskonzentration eingeschränkt? Wird hier der Ausnahmezustand bewusst genutzt, um ein Regime der „Biosicherheit“ (S. 96) zu errichten?

Agambens radikale Kritik richtet sich aber nicht nur gegen eine Politik der „Biosicherheit“, sondern auch gegen die Medizin, ja gegen die Wissenschaft als solche. In „Die Medizin als Religion“ postuliert er, diese sei, neben Christentum und Kapitalismus – Walter Benjamin steht wohl nicht nur für diesen Gedanken Pate – zur Religion geworden (S. 77). Sie unterwerfe den Menschen durch Dogma und Kultus. Indem sie den Menschen auf seine Biologie, auf seine „rein vegetative Substanz“ reduziere (S. 61) und sie in einen Gegensatz zur Krankheit einschreibe, entwerfe die Medizin ein quasi gnostisch-dualistisches Glaubenssystem, das mit Hilfe kultischer Handlungen (etwa Medikamentengabe, Operationen etc.) durch Zwang aufrechterhalten wird (S. 80–82). Agamben zieht daher den Schluss: „Hier [in der Bekämpfung der Pandemie, Anm. LJ] handelt es sich um eine kultische Praxis und nicht um ein rationales wissenschaftliches Erfordernis, wie unmittelbar einleuchtet“ (S. 81).

Einen Ausweg aus dem vermeintlich mithilfe der Pandemie eingeschlagenen Weg des Ausnahmezustands erkennt Agamben in der titelgebenden Frage: „An welchem Punkt stehen wir? Diese Frage gilt es so gut wir können zu beantworten wo immer wir sind, aber in jedem Fall mit unserem Leben und Handeln, und nicht nur mit Worten“ (S. 33): Agamben fordert im gleichnamigen Beitrag die Besinnung auf eine alternative Lebensweise ohne Fernreisen, Konsumüberfluss und mit einer Stärkung des öffentlichen Gesundheitswesens (S. 31–33). Hier werden in leisen Tönen positive Seiten seiner Kritik erkennbar. Angesprochen auf konkrete Alternativen zur derzeitigen Pandemiepolitik der europäischen Staaten bleibt Agamben hingegen stumm und flüchtet sich ins Abstrakte. Die Kritik muss radikal bleiben – um den Preis der Alternativlosigkeit (S. 102f.). Er versteht Kritik so radikal, dass nichts Positives stehenbleiben darf.

Innerhalb dieser Fundamentalkritik der wissenschaftlichen, praktischen, wie politischen Bekämpfung der Pandemie erscheint es dann auch nur noch wenig verwunderlich, wenn der gefeierte wie kritisierte Philosoph die Grenzen zur Verschwörungsideologie mal mehr, mal weniger bewusst zu verwischen sucht. Nicht nur sei die Pandemie eine Erfindung, auch Bill Gates und dessen Engagement für die globale Gesundheitspolitik finden auf geradezu verstörende Weise Erwähnung.

Agamben bietet in den versammelten Texten radikale Kritik an Politik und Wissenschaft. Diese trifft oftmals kaum den kritisierten Gegenstand oder muss ihn herbeibeschwören. Dennoch darf nicht übersehen werden, dass viele Texte genau in dieser Haltung auch eine aufrüttelnde Stärke besitzen. Sie sind als Kritik im Sinne Foucaults zu lesen. In „An welchem Punkt stehen wir?“ sind kritische Interventionen und durchaus ethisch-philosophisch anregende Texte zu finden. Sie fragen, welchen Preis wir zu zahlen bereit sind, um Gesundheit und Leben zu schützen. Sie fragen nach den politischen Grundstrukturen, die in der Pandemie entstanden oder verschwunden sind. Die Texte verbinden Fragen der Public-Health-Ethik mit denen radikal-demokratischer Theorie, übersehen dabei jedoch in ihrer Radikalität oftmals die Ambivalenz der Ereignisse und immunisieren sich selbst gegen Kritik. Für den Wunsch, nicht so und nicht um diesen Preis regiert zu werden, scheint Agamben jedes Mittel, auch das der Verschwörungsideologie, recht.
